# Management of Neuropsychiatric and Sleep Symptoms in LBD

**DOI:** 10.1002/alz.090529

**Published:** 2025-01-09

**Authors:** Chiadi U. Onyike

**Affiliations:** ^1^ Johns Hopkins University School of Medicine, Baltimore, MD USA

## Abstract

The presentation of neuropsychiatric and sleep features in Lewy body dementia (LBD) are challenging to identify and manage. The US Based Diamond Lewy Neuropsychiatric Symptoms management guidelines provide expert consensus recommendations for the assessment and treatment of the neuropsychiatric features of LBD. These guidelines address the management of delusions, hallucinations, agitation and aggression, apathy, depression and anxiety. Pharmacologic interventions will be discussed in detail, in order to describe recommended agents, prescribing strategies, and potential adverse effects and safety aspects. The parallel Diamond Lewy Sleep Disturbances management expert guidelines will also be discussed, to bring to light best practices in the assessment and management of excessive daytime sleepiness, restless legs syndrome (RLS), motor‐related sleep disturbances, sleep apnea, REM sleep behavior disorder, insomnia and sleep fragmentation.

